# Adaptation strategies and neurophysiological response in early-stage Parkinson's disease: BioVRSea approach

**DOI:** 10.3389/fnhum.2023.1197142

**Published:** 2023-07-17

**Authors:** Deborah Jacob, Lorena Guerrini, Federica Pescaglia, Simona Pierucci, Carmine Gelormini, Vincenzo Minutolo, Antonio Fratini, Giorgio Di Lorenzo, Hannes Petersen, Paolo Gargiulo

**Affiliations:** ^1^Institute of Biomedical and Neural Engineering, Reykjavik University, Reykjavik, Iceland; ^2^Department of Engineering, University of Campania L. Vanvitelli, Aversa, Italy; ^3^Department of Electrical, Electronic and Information Engineering, University of Bologna, Cesena, Italy; ^4^Department of Pharmacy and Biotechnology, University of Bologna, Bologna, Italy; ^5^Department of Civil Engineering and Computer Science Engineering, Tor Vergata University of Rome, Rome, Italy; ^6^Engineering for Health Research Centre, Aston University, Birmingham, United Kingdom; ^7^Laboratory of Psychophysiology and Cognitive Neuroscience, Department of Systems Medicine, Tor Vergata University of Rome, Rome, Italy; ^8^IRCCS Fondazione Santa Lucia, Rome, Italy; ^9^Department of Anatomy, University of Iceland, Reykjavik, Iceland; ^10^Department of Science, Landspitali University Hospital, Reykjavik, Iceland

**Keywords:** postural control, early-stage Parkinson's disease, quantitative neurophysiology, BioVRSea, balance control

## Abstract

**Introduction:**

There is accumulating evidence that many pathological conditions affecting human balance are consequence of postural control (PC) failure or overstimulation such as in motion sickness. Our research shows the potential of using the response to a complex postural control task to assess patients with early-stage Parkinson's Disease (PD).

**Methods:**

We developed a unique measurement model, where the PC task is triggered by a moving platform in a virtual reality environment while simultaneously recording EEG, EMG and CoP signals. This novel paradigm of assessment is called BioVRSea. We studied the interplay between biosignals and their differences in healthy subjects and with early-stage PD.

**Results:**

Despite the limited number of subjects (29 healthy and nine PD) the results of our work show significant differences in several biosignals features, demonstrating that the combined output of posturography, muscle activation and cortical response is capable of distinguishing healthy from pathological.

**Discussion:**

The differences measured following the end of the platform movement are remarkable, as the induced sway is different between the two groups and triggers statistically relevant cortical activities in α and θ bands. This is a first important step to develop a multi-metric signature able to quantify PC and distinguish healthy from pathological response.

## 1. Introduction

Parkinson's Disease (PD) is a progressive disorder of the nervous system characterized by muscle tremors, muscle rigidity, decreased mobility (bradykinesia), stooped posture, slow voluntary movements, and a mask-like facial expression. It may take time to diagnose because some of its symptoms are associated with the natural process of aging (Tolosa et al., 2006). Globally, disability and death in PD are increasing faster than any other neurological disorder. The World Health Organization (WHO) reports that the prevalence of PD has doubled in the past 25 years and world estimates count over 8.5 million individuals with PD in 2019. In people with early-onset PD, the initial symptoms can arise between the ages of 21 and 40 years, while the first symptoms in juvenile-onset disease occur before the age of 20 years. Nowadays, a standard criterion in the evaluation of PD is still one of the main goals for clinicians. Finding the right category for the progression of the disease is necessary to prescribe the best treatment. Specific signs, symptoms, or test results can help in the classification of the disease. Over the years, accuracy has been improved by new diagnostic protocols that consider qualitative and quantitative aspects (Maffoni et al., 2017; María et al., 2020). Defining early-stage Parkinson's subjects when the symptoms are silent or weak remains a challenge.

**Figure 1:**
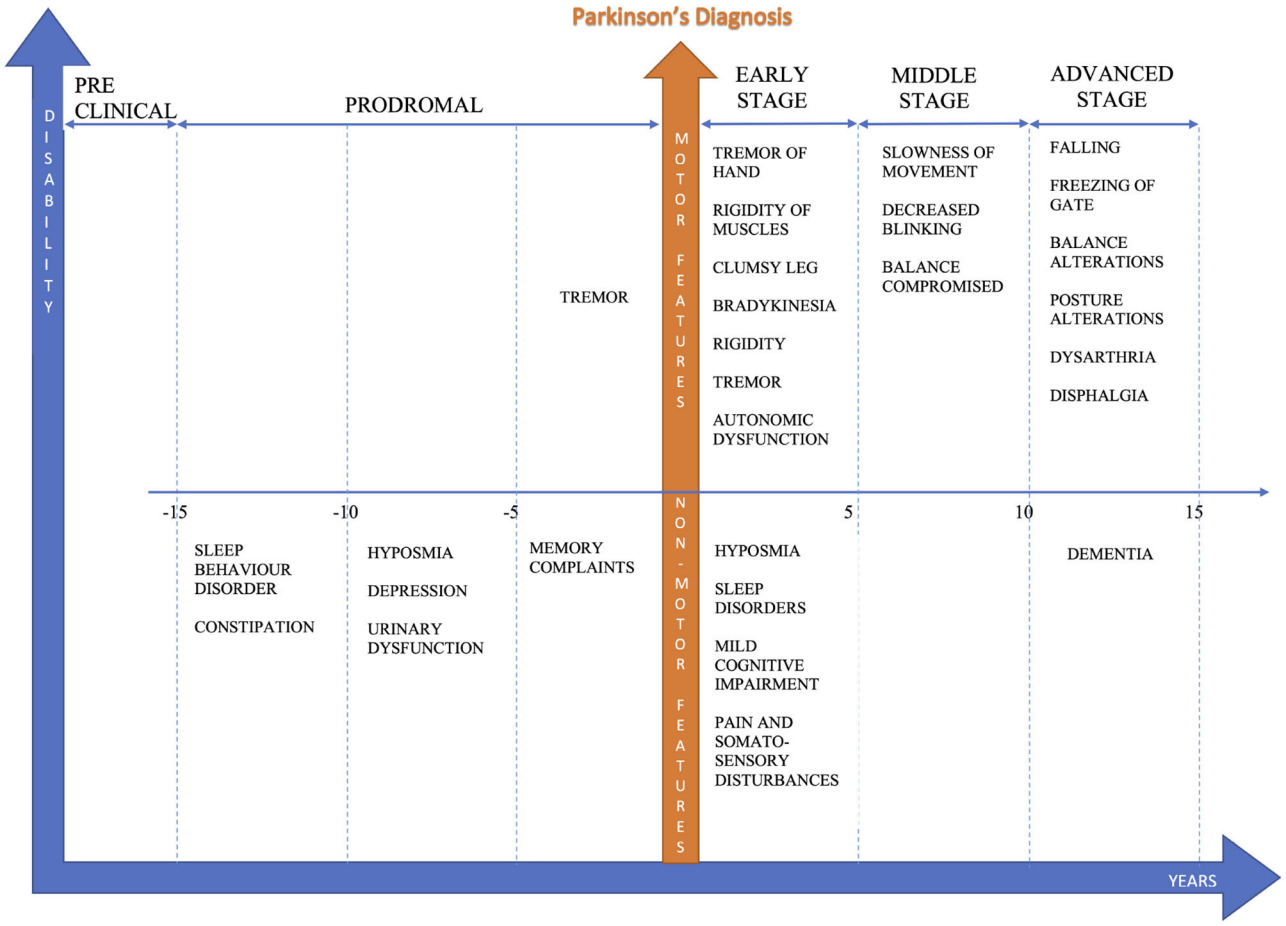
Symptoms and stages before-after Parkinson's diagnosis.

PD stages are identified based on clinical observations: according to the Hoehn-Yahr staging system, stages are based primarily on motor symptoms (Goetz et al., 2007). Pre-clinical is characterized by the absence of signs or symptoms - genetic testing and counseling are available to identify risk factors. Prodromal corresponds to a stage of neurodegenerative changes. Symptoms are unspecific but the identification of early changes allows to intervene with initial therapies. Early-stage symptoms include mild tremors and some walking difficulty. It can affect only one side of the body and produce a decrease of facial expressions. These symptoms do not interfere with daily life much and are not always obvious (Goetz et al., 2007). Middle-stage balance and coordination are affected: a moderate-to-severe disability that affects daily life (Goetz et al., 2007). Later-stage subjects have difficulty standing and walking even with aids. Patients in this stage have severe disability (Goetz et al., 2007). Figure 1 reports the general classification of PD stages according to its main symptoms.

**Figure 2:**
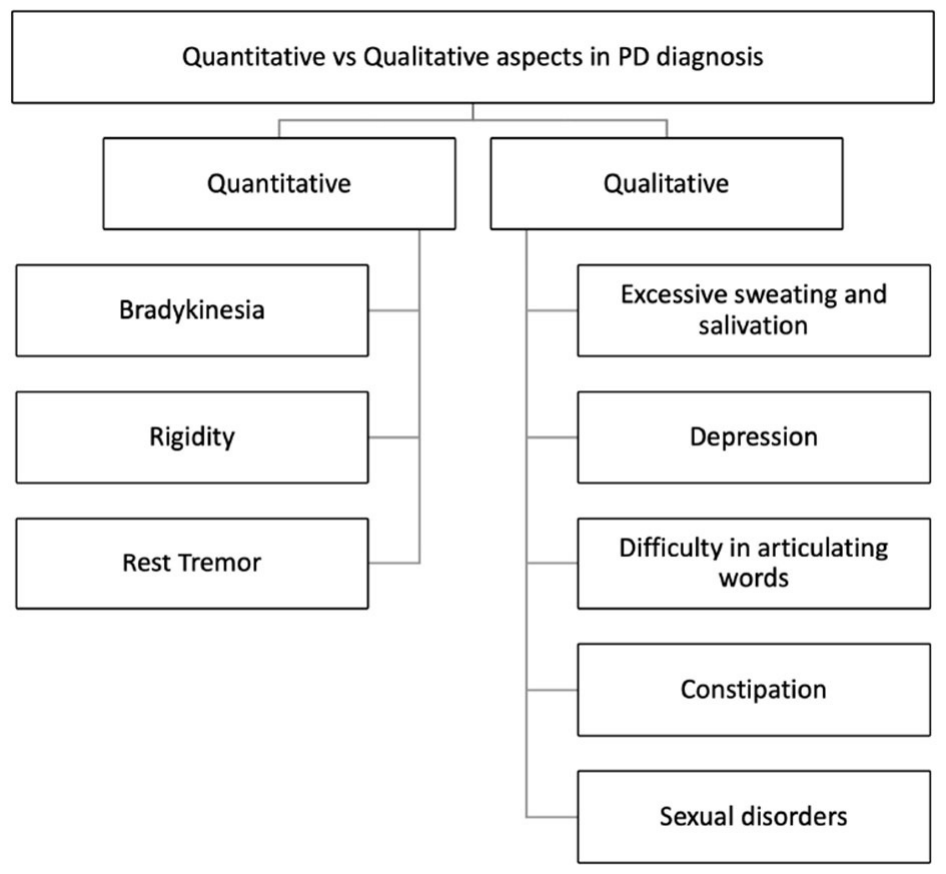
Quantitative and qualitative aspects in Parkinson's Disease diagnosis (Maffoni et al., 2017).

A preliminary analysis can be carried out by clinicians taking into account qualitative and quantitative aspects of PD, as shown in Figure 2.

Gait mobility and gait impairment is also seen to evolve with the progression of the disease are included as parameters of investigation. Issues with gait initiation, freezing of gait, reduced balance, and difficulties in postural control (Martignon et al., 2021) are some of common symptoms. Table 1 summarizes the main tests used in mobility and gait analyses.

**Table 1:** Gold standard tests for assessing gait ability.

**Assessment**	**Test**	**Parameter investigated**
Balance	Timed Up and Go Test	Functional mobility
	Tinetti Balance and Gait Test	Static and dynamic balance
	Retropulsion Test	Postural stability
	One-leg Stance	Static balance
	Åstrand-Rhyming protocol during Graded Exercise Test	Dynamic balance
	Balance Evaluation System Test	Balance systems
	Mini Balance Evaluation Systems Test	Dynamical balance
	Berg Balance Scale	Static and dynamic balance
Endurance	2-min Walk Test	Functional capacity, walking ability
	6-min Walk Test	Physical capacity and gait
	Two-minute Step Test	Aerobic capacity
	Modified Bruce Protocol during Graded exercise test	Cardiac functional capacity
	Åstrand-Rhyming protocol during Graded exercise test	Maximal functional capacity
	Borg Ratio Scale	Physical capacity
Resistance	Handgrip Strength Test	Upper limb strength
	Isokinetic Strength Test	Upper/lower limb strength
	Manual Muscle Test	Individual or grouped muscle strength
	Arm Curl Test	Upper limb strength
	Chair Stand Test	Lower limb strength
	Five Time Sit to Stand Test	Lower limb strength
	One Repetition Maximum Test	Maximum lower or upper limb isotonic
Flexibility	Goniometer	General joint flexibility
	Inclinometer	Angles of slope measurement
	Leighton flexometer	Joint flexibility
	Sit and Reach Test	Lower back and hamstring muscles tightness
	Back Scratch Test	Shoulder range of motion

Among the innovative techniques, blood tests show potential to be used for the detection of early-stage PD. Researchers identified a molecular profile that defines the disease but it is still under investigation and not yet available clinically (Agliardi et al., 2021).

In recent years, non-invasive brain imaging techniques have become more accurate for the detection of differences in brain morphology and functional activities in Parkinson's subjects (Politis, 2014). Brain positron emission tomography (PET) can estimate the disease progression and can be used to confirm the clinical diagnosis of PD. With specific radioactive drugs (18F-DOPA and 18-FDG) absorbed into the bloodstream, PET can provide very precise brain region and activation in PD subjects (Lu and Yuan, 2015). Single positron emission tomography (SPECT) is also used as method to confirm a Parkinson's diagnosis by highlighting cerebral blood flow and dopamine transporters in the brain (Lu and Yuan, 2015). In patients with Parkinson's disease, a distinct intensity pattern can be noted in the brain region that is deeply affected by degeneration, namely the basal ganglia that controls movement. Magnetic resonance imaging (MRI) is also used to diagnose PD in early onset subjects. MRIs can show small changes and damage in the brain tissue that can indicate PD. Often, these markers are present even before symptoms of PD begin. Transcranial sonography (TCS) has been established as a valuable supplementary tool in the diagnosis of PD. Alterations in the area of the hypoechogenic mesencepahlic brainstem can be visualized in about 90% of PD patients, which is measured planimetrically to determine the magnitude of the change (Berg et al., 2008). Increased iron levels contribute to this sonographic abnormality and indicate that iron can be responsible for the change in the echo signal. Iron accumulation can be a very early indicator in the pathogenesis of PD. Electroencephalography (EEG) can detect damage in the central nervous system and alterations in neurophysiological activity associated with PD. In recent studies, quantitative analysis of EEG data identified significant differences in PD patients versus healthy subjects. In particular, the anterior cingulate and temporal lobe are areas with an established pathology in PD. Changes in cortico-cortical and cortico-thalamic coupling were observed as excessive EEG beta coherence in PD patients (Waninger et al., 2020).

Map structure and functions of the brain are obtained measuring the signals produced by neural activity. Each region can have a particular influence according to the disease and the activation of an area can be considered important in the understanding of the progression of the disease. Although cortical EEG coherence can serve as a reliable measure of disease severity, the use of EEG to study PD has not been fully investigated. Neurophysiological signals provide instantaneous information and can aid in improving the accuracy of the diagnosis.

EEG signals have different specific frequency bands. Features in sub-bands are particularly important to characterize different brain states. The standard frequency bands of interest are δ-band (0–4 Hz), θ-band (4–8 Hz), α-band (8–13 Hz), and β-band (13–30 Hz). Moreover, the quantification of EEG rhythms could provide an important biomarker for different neuropsychiatric and neurological disorders, such as schizophrenia, Alzheimer's disease, epilepsy, and Parkinson's disease (Hampel et al., 2010; Gandal et al., 2012; Kheiri et al., 2012). The combination of new analysis methods and EEG signal processing can contribute to the detection of early-stage PD. EEG reveals more important information underlying brain dysfunctions, which would be lost if analysis were restricted to traditional methods. Nowadays, many novel methods are suggested for EEG signal processing.

A recent study analyzed the EEG signals from 15 early-stage PD patients and 15 age-matched healthy controls during eyes-closed resting state (Han et al., 2013). Most EEG electrodes showed an increase in θ-band relative power for PD patients, while several other electrodes decreased, such as in the frontal and occipital cortex (Fp1, Fp2, F7, F3, Fz, Oz). Moreover, an increase in δ-band relative powers were reported, and a decrease in α-band and β-band relative powers for PD patients compared with healthy patients. Other studies present higher spectral power in the low frequency domain of EEG, compared with controls. Also in these cases, subjects were in the resting awake condition with the eyes closed (Moazami-Goudarzi et al., 2008; Serizawa et al., 2008).

Postural control (PC) and adaptation are part of a complex system to maintain or restore balance from any position or during motor activity. The central nervous system is fundamental in PC strategies and electroencephalography can underline the different cortical brain activities under different postural perturbations (Mochizuki et al., 2010; Barollo et al., 2020). PD usually interferes in this regulatory system, as can be clearly demonstrated by most motor symptoms, but to date, no study has yet been conducted on the analysis of postural kinematics in movement disorders. Our aim is to (i) investigate the postural strategy adopted in PD individuals and in healthy subjects; (ii) describe adaptation and how the brain adapts to the induced movement of a platform and visual stimuli using virtual reality (VR). However, postural control and adaptation have been extensively studied in healthy and blind subjects. In a recent study (Barollo et al., 2020), postural kinematics from HD-EEG have been measured during a postural perturbation applied to calf muscles. The main changes in cortical activity were found in Absolute Spectral Power (ASP) over four frequency bands. For postural adaptation, increases in the θ band in the frontal-central region for closed-eyes trials, and in the θ and β bands in the parietal region for open-eyes trials were reported. In habituation of the stance, no significant variations in ASP were observed during closed-eyes trials, whereas an increase in the θ, α, and β bands were observed with open eyes (Curtis et al., 2001). Furthermore, open-eyed trials generally yielded a greater number of significant differences across all bands during both adaptation and habituation, suggesting that cortical activity during postural perturbation may be regulated with visual feedback. This clearly shows a correspondence in cortical activity and postural kinematics during postural perturbation, and could also be developed for pathological postural control.

**Figure 3:**
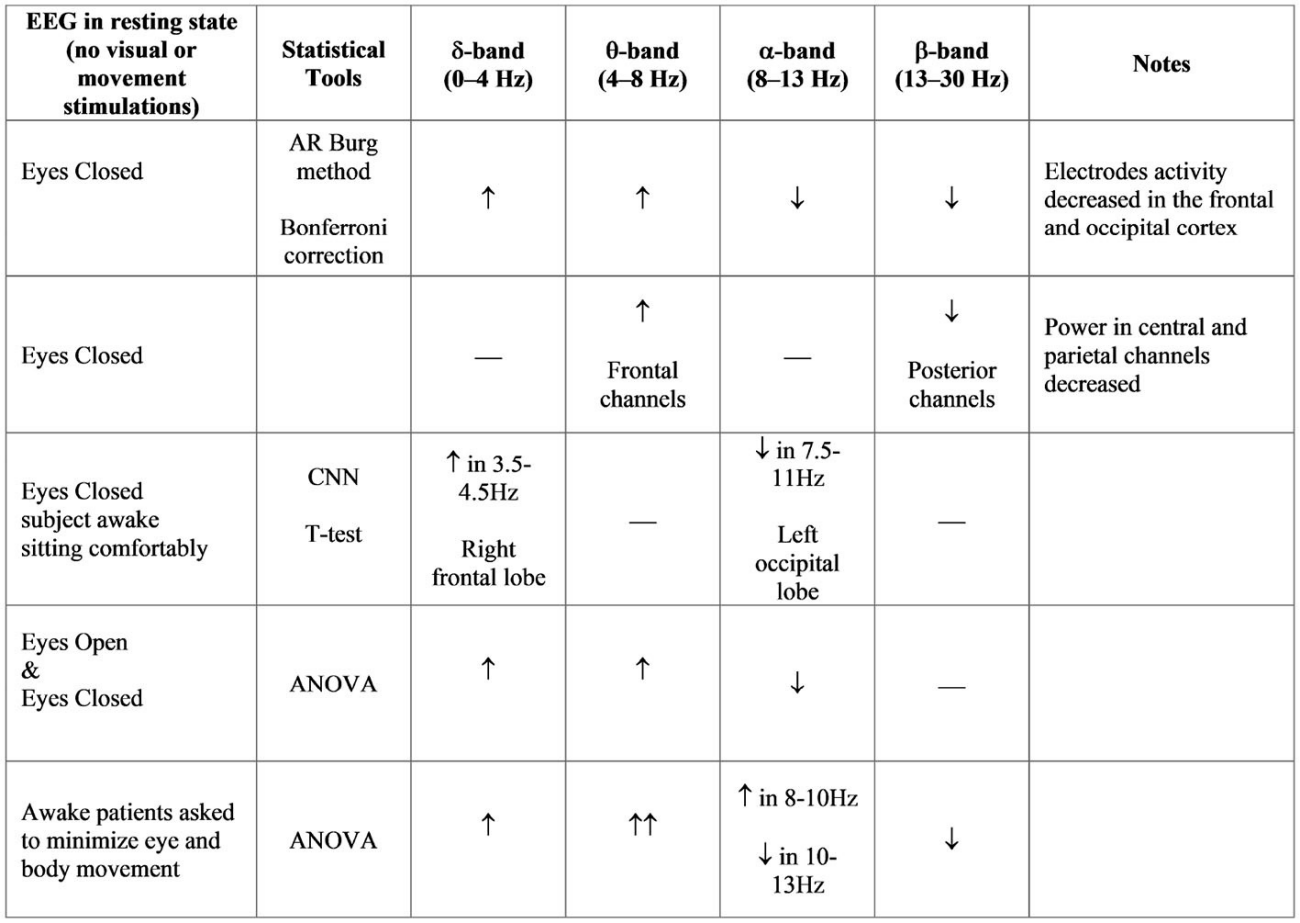
ASP bands analysis in different studies (Neufeld et al., 1988; Sinanović et al., 2005; Bosboom et al., 2006; Han et al., 2013; Chu et al., 2021).

Other studies show similar results in healthy subjects, suggesting cortex activity as the main change in the frontal-central and frontal-parietal cortical regions during balance perturbation, specifically within α and θ frequencies (Sipp et al., 2013; Hülsdünker et al., 2015). Moreover, the increase of the ASP in the central region is demonstrated during high-demand postural correction, such as balance maintenance without allowing corrective foot placement (Barollo et al., 2020). In accordance, the increase in θ activity in the frontal-central regions implies the processing of postural stability during balance control. Thus, ASP differences in the θ band signify the planning of corrective steps and the analysis of the consequences of the subject falling. Instead, the significant differences in the α band reflect an inhibition of error detection within the cingulate cortex due to habituation. Other studies have been carried out with the blind (including both congenital blindness and acquired blindness). Congenitally blind subjects had poorer postural control (anterior-posterior and medio-lateral body swing) compared to sighted subjects. They use a more efficient mechanism for maintaining balance control through joint stiffness. These findings demonstrate that motor coordination, localization, or perception of body segments and movements in visually impaired individuals can be compensated by enhancing the proprioceptive and vestibular systems. Blindness leads to impaired postural balance and imbalance in static and dynamic tasks (Parreira et al., 2017). An overview of EEG studies in postural control is provided in Figure 3.

**Figure 4:**
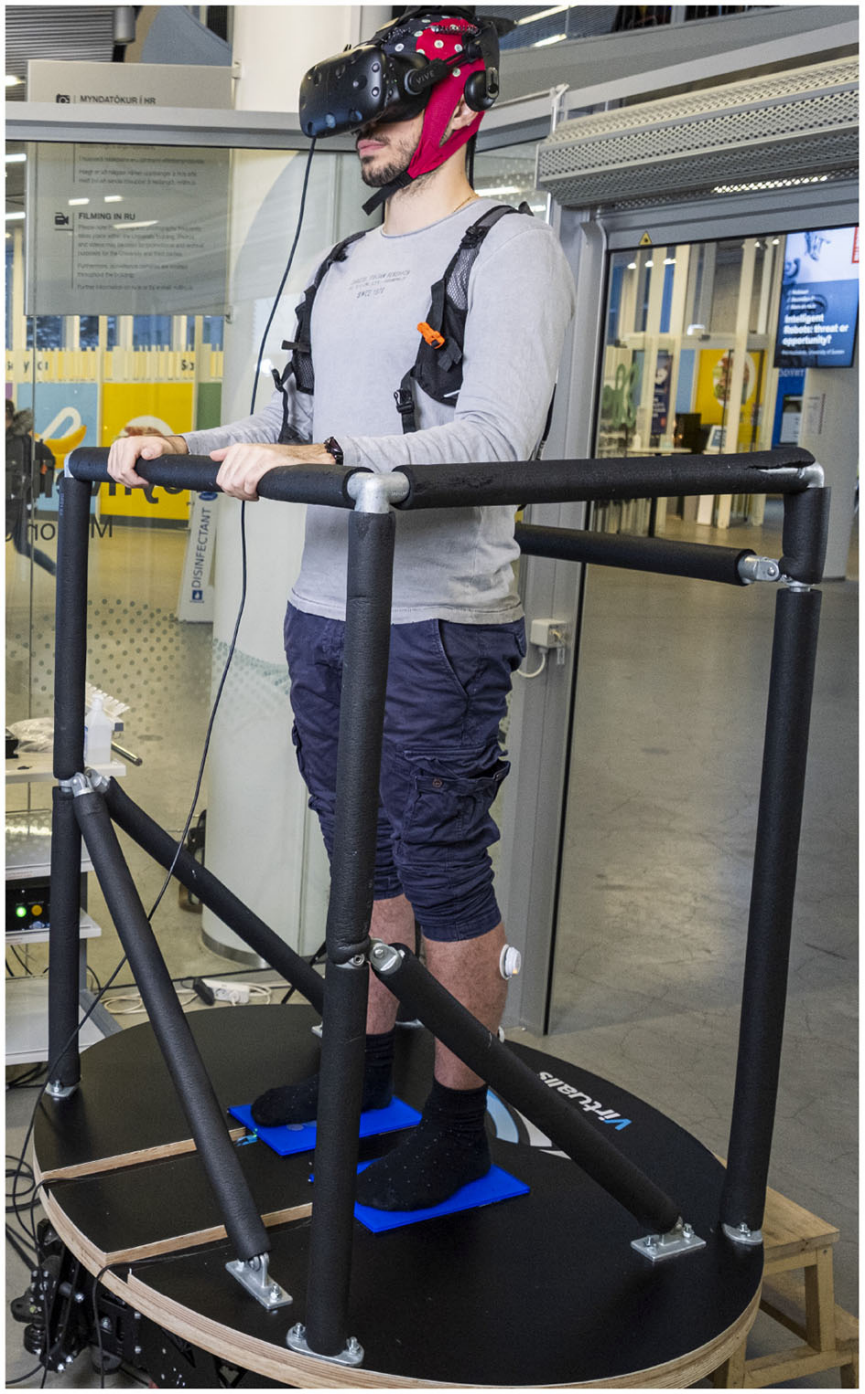
BioVRSea experimental setup.

Our novel BioVRSea setup introduces a unique multi-biometric system that combines virtual reality and a moving platform to evaluate the postural control response. The system is designed to imitate the sensation of being at sea on a small boat, a situation which involves different balancing strategies. During the experiment, there are six phases (see Table 2, Figure 4) in which different biosignals are measured such as electromyography (EMG), center of pressure (CoP), and electroencephalography (EEG). Some of our recent studies emphasize the importance of BioVRSea setup allowing cohort differentiation and pathology assessment (Recenti et al., 2021; Aubonnet et al., 2022). The advantage of using BioVRSea is that we are measuring quantitative signals associated with postural control in a challenging environment. The experiment is a prototype and the purpose of the current research with BioVRSea is to gather as much data as possible with many simultaneous measurements in order to extrapolate the most relevant features which could then be used in a clinical setting, with a lower-profile machine that could be accessed easily by those with mobility problems. Current diagnosis of PD relies primarily on the presence of motor symptoms in the patient (such as MDS diagnosis criteria (Postuma et al., 2015) and generally lacks any quantitative measurement such as we perform in the BioVRSea experiment.

**Table 2:** BioVRSea experimental paradigm.

**Time (s)**	**Segment**	**VR Scene**	**Position of Hands**	**Platform**
0-120	Baseline	Mountains	By side	Stationary
120-160	PRE	Sea	By side	Stationary
160-200	25%	Sea	On bars	Moving
200–240	50%	Sea	On bars	Moving
240-280	75%	Sea	On bars	Moving
280-320	POST	Sea	By side	Stationary

In this paper, candidates with early-stage Parkinson's Disease undergo a postural control task in the BioVRSea environment. We focus on the differences between the two cohorts in the PRE and POST phases. Our focus on PRE and POST will allow the study of early stage PD while they are adapting to a motor stimulus.

## 2. Materials and methods

### 2.1. Participants

Nine early-stage (recently diagnosed) PD participants (6 male, 3 female, between 56 and 76 years of age) and 29 healthy subjects (17 male, 12 female, between 50 and 73 years old) took part in the BioVRSea experiment. Few of them showed physical evidence of early stage PD such as weak tremor or onset of postural instability. All were taking the drug Levodopa as part of their treatment.

### 2.2. BioVRSea experiment

A 64-channel wet EEG was used record brain response to VR and motion stimulation. Wireless EMG sensors were placed on the tibialis anterior (TA), gastrocnemius lateral (GL), and soleus (S) muscles of both legs. A heart rate sensor strapped around the chest. For the experiment, the participant were asked to stand onto the force plates embedded in the platform. Finally, the participants donned the VR goggles. The experimental protocol was then explained to the participant. Participants stood quietly on the platform with their hands by their side observing a mountain view for the first 2 minutes of the experiment (Baseline). Then, the scene in the VR goggles changed, beginning the sea simulation but no platform movement. The participants remained standing quietly with their hands by their side for the first 35 seconds of the sea simulation (PRE). After the PRE phase the platform began synchronized movement with the sea scene in the VR goggles, increasing from 25% to 75% of maximal wave amplitude. For a total of 120 seconds the participants held the bars of the platform while continuing to observe the sea simulation. Finally, the platform stopped moving while the sea simulation is still showing and the participant was asked to remove their hands from the bars and attempt to stand quietly with their hands by their side for the final 40 seconds of the experiment. This is called the POST phase of the experiment; it is performed identically to the PRE phase but after the participant has performed movement in the central part of the procedure. A table of the VR experiment protocol is shown below in Table 2, shows a schematic of the experimental setup. Each participant took part in a single trial according to the experimental protocol.The subject undergoes different stimuli: visual stimulus (PRE-phase), motor stimulus (movement phase), and balance control (POST-phase).Measured data was post-processed in Matlab and analysis was made in the PRE and POST phases of the experiment. Each analysis pipeline for a particular measurement is explained below.

#### 2.2.1. Heart rate

Heart rate was measured using a chest heart sensor (Polar Electro, Kempele, Finland, sampling frequency 1 Hz). The average and standard deviation for the HR for each section of the experiment was calculated.

#### 2.2.2. EMG analysis

Muscle electrical activities from the lower limbs were acquired using six wireless EMG sensors (sampling frequency of 1600 Hz) placed on the tibialis anterior (TA), gastrocnemius lateral (GL), and soleus (S) muscles of each leg (Kiso ehf, Reykjavik, Iceland). EMG data processing was performed using Matlab 2021b. EMG data were filtered using a 4th-order Butterworth filter. Seven features were computed in the frequency domain and thirty-six features in the time domain for each muscle and each phase of the experiment. These features are listed in **Table 5**.

##### 2.2.2.1. Statistical analysis

The Shapiro-Wilk test along with visual inspection of the distribution of each variable were used to test the normality of the data. Statistical comparisons between the healthy and PD groups in both the PRE and POST phases were carried out using the t-test with Welch's correction for the normally distributed variables and the Mann-Whitney U-test for the non-normally distributed variables, with a significance value of *p* < 0.05. Effect sizes were calculated through the non-parametric Cliff's delta using the R package “effsize” (Torchiano, 2020). Cliff's delta ranges from +1 if all observations in the first group are larger than all observations in the second group, to -1 if all observations in the first group are smaller than all observations in the second group (Cliff, 1993).

#### 2.2.3. CoP analysis

**Figure 5:**
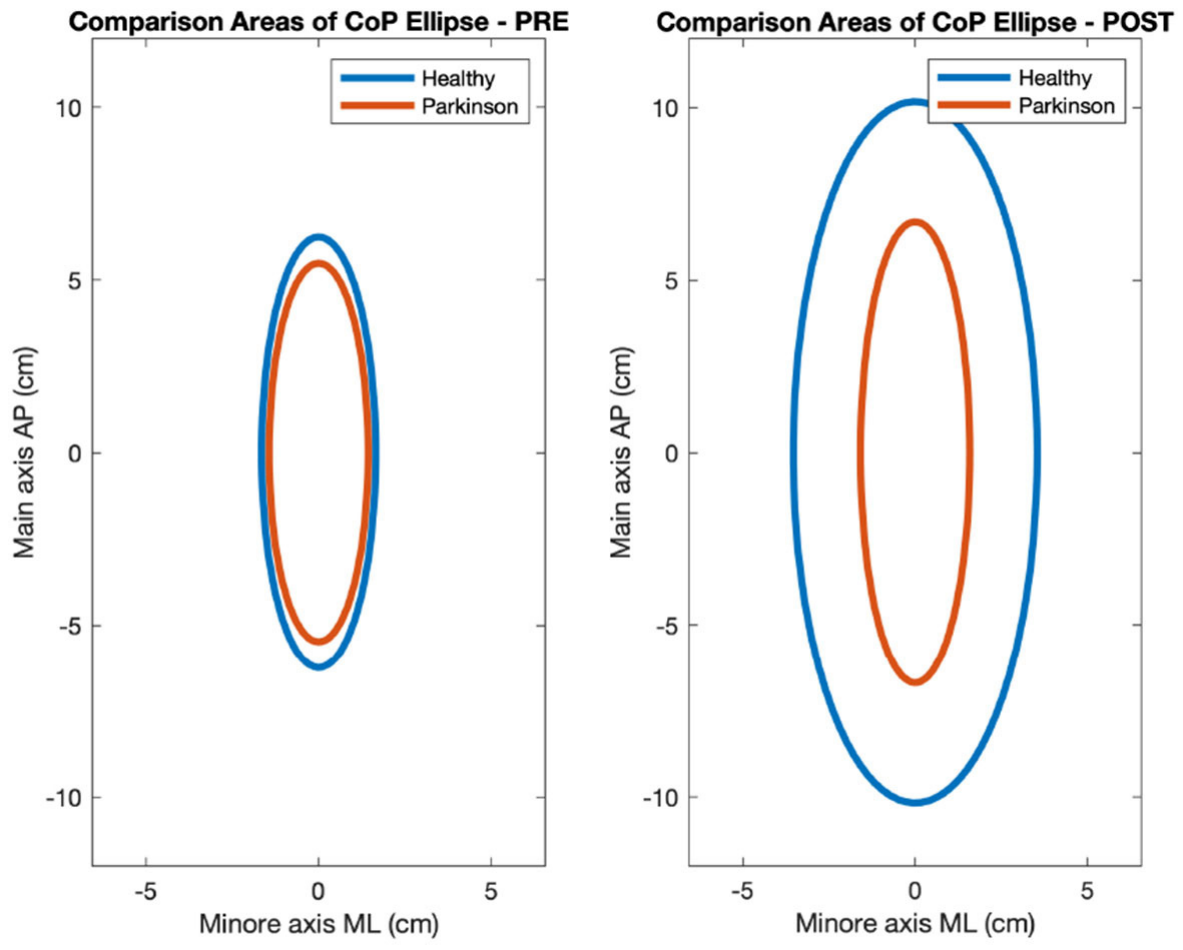
Ellipse areas comparison between Parkinson and healthy subjects in PRE-POST phases.

CoP measurements were made using 4 sensors located under each foot platform. The sensors give information about the center of mass in the Antero-Posterior and Medio-Lateral axis (Virtualis, Clapiers, France, sampling frequency 90Hz). The processing of the CoP data was performed using Matlab 2021b. During the experiment, the force platform records the movement of the Centre of Pressure (CoP), a projection of the center of mass of the subject on the plane of the machine, also called stabilogram. The CoP data was filtered with a Savitsky-Golay filter with window size 7. Included in the CoP analysis were a number of multi-scale entropy measurements, which have been shown to have great importance in the analysis of CoP data in discriminating between pathological subjects (Busa and Emmerik, 2016). Multi-scale entropy measurements include features such as complexity index (CI), which indicate the complexity of the CoP signal as calculated using multi-scale entropy methods. We extract several parameters from the stabilogram for evaluating the postural control response of the subject during the experiment. The list of features extracted from the CoP is outlined in Table 3. Statistical analysis normality was checked through the Shapiro-Wilk test and visual inspection of the variables, and comparisons were made between the healthy and PD groups for all features in the PRE and POST phases using the t-test with Welch's correction and the Mann-Whitney U-test with significance level *p* < 0.05. Effect sizes were calculated using Cliff's delta. Sway profiles were also outlined using 95% confidence ellipses in PRE and POST, as seen in Figure 5.

**Table 3:** CoP features calculated - bold shows the features that were significantly different in the POST phase of the experiment when comparing the PD and healthy groups.

**Feature**	**PRE *p*-value**	**POST *p*-value**	**Cliff delta**
Consecutive movement samples on the support plane (TOTEX)	-	-	0.379
Consecutive movement samples on ML plane(TOTEX-ML)	-	-	0.402
Consecutive movement samples on AP plane(TOTEX-AP)	-	-	0.371
Square root distance between a point and the plane origin (RD)	-	0.0173	0.494
**Mean distance in medio-lateral direction (MDIST-ML)**	-	0.0339	0.494
**Mean distance in antero-posterior direction (MDIST-AP)**	-	0.0173	0.494
Mean velocity on support plane (MVELO)	-	-	0.379
Mean velocity on ML plane (MVELO-ML)	-	-	0.402
Mean velocity on AP plane (MVELO-AP)	-	-	0.371
**Root mean square distance respect to origin (RDIST)**	-	0.0115	0.540
**Root mean square distance in medio-lateral direction (RDIST-ML)**	-	0.0256	0.517
**Root mean square distance in antero-posterior direction (RDIST-AP)**	-	0.0115	0.533
**Medio-lateral sample entropy (ML-SampEn)**	-	0.0002	-0.709
Antero-posterior Sample Entropy (AP-SampEn)	-	-	-0.333
**Medio-lateral complexity index (ML-CI)**	-	0.0009	-0.793
Antero-posterior Complexity Index (AP-CI)	-	-	-0.325
**Ellipse area**	-	0.0083	0.571
**Ellipse angle**	-	-	0.057
**Ellipse main axis length**	-	0.0067	0.571
**Ellipse minor axis length**	-	0.0127	0.571
**Standard deviation in antero-posterior direction (SD AP)**	-	0.0115	0.532
**Standard deviation in medio-lateral direction (SD ML)**	-	0.0257	0.517
**SD magnitude**	-	0.0074	0.563
SD direction	0.0128	-	-0.256
**Magnitude entropy**	-	-	-0.510
**Direction entropy**	-	-	-0.249
**Multivariate complexity index (multivariate CI)**	-	0.0128	-0.639
Antero magnitude	-	-	0.379
Antero angle	-	-	0.019
**Postero magnitude**	-	0.0141	0.548
**Postero angle**	-	-	0.065
Left magnitude maximum	-	0.0406	0.417
Left angle	-	-	-0.065
**Right magnitude maximum**	-	0.0282	0.448
**Right angle**	-	-	-0.494

#### 2.2.4. EEG analysis

The CA-204-64 wet electrode cap, EegoTM mylab with sampling frequency of 4096 Hz, measured the brain electrical activity in 64 electrodes. Raw EEG signals were processed using Matlab 2022b, Brainstorm, EEGlab 2022.1 and Automagic toolboxes (Pedroni et al., 2019). The signals were divided in segments for each phase of the experiment, then the signals were down-sampled from 4,096 to 1,024 Hz. During pre-processing, different settings were applied to the EEG, such as ICA MARA artefact removal and high pass and low pass filters respectively set at 1 Hz and 45 Hz. The data were interpolated finding the locations of bad channels. The EEG data set can be displayed as electrode channel plots, allowing a quick overview of data quality. Then, the absolute power spectral density (PSD) was calculated and compared between PD and Healthy groups in each phase of the experiment for each of the delta, theta alpha and delta bands. A Mann-Whitney U-test with significance level (α = 0.05) was used to determine significance. False detection rate (FDR) correction was applied to each electrode.

## 3. Results

The following results are reported for the analysis of the PRE and POST phases of the experiment with the aim of distinguishing between the PD and healthy groups based on their biosignal responses. Our experiment was able to identify changes in many of the analyzed domains.

The protocol is a visual-motor simulation of being on a boat and part of the subjects experienced the feeling of seasickness. Just under half of PD subjects experienced actual discomfort with various symptoms (reported on questionnaires), and a smaller percentage of them reported a self-assessment of motion sickness in daily life.

### 3.1. Heart rate

The results of the heart rate analysis are shown in Table 4. An increase in beats per minute (bpm) was measured in PD subjects, although not statistically significant. No statistically significant differences were found between groups.

**Table 4:** Average and standard deviation for Heart Rates inside cohorts in PRE and POST phases.

**Beats per minute (BPM)**	**Parkinson's**	**Controls**
PRE-phase	88.76 ± 18.09	82.48 ± 14.63
POST-phase	94.19 ± 20.97	82.54 ± 14.35

### 3.2. EMG

The right TA muscle showed a number of statistically significant features in the POST phase, with a p value (*p* < 0.05) and the corresponding effect sizes for each variable shown in bold in Table 5. The right side could be considered the dominant leg in the prevalence of the group. Significant changes were found also in the left soleus (MN - mean, *p* = 0.003, cliff delta = -0.64) in the POST phase and the soleus right which had one significant feature (MD -median, *p* = 0.007, cliff delta = -0.586) in the PRE phase.

**Table 5:** EMG features—bold shows features that were significantly different between PD and healthy groups in the right tibialis anterior muscle in the POST phase.

**Feature**	**PRE *p*-value**	**POST *p*-value**	**cliff delta POST**
Total Power (PT)	-	-	0.425
**Maximum Power (Pmax)**	-	0.0394	0.464
Maximum frequency (Fmax)	-	-	0.141
Median Frequency (FMD)	-	-	0.153
Mean frequency(FMN)	-	-	0.191
Frequency Kurtosis (Fkurt)	-	-	0.073
Frequency skewness (Fskew)	-	-	0.080
**Average amplitude change (AAC)**	-	0.0256	0.502
Average energy (AE)	-	-	0.425
**Absolute value of the summation of the exponential root(ASM)**	-	0.0195	0.524
**Absolute value of the summation of the square root (ASS)**	-	0.0234	0.0509
Coefficient of variation (CV)	-	-	-0.172
**Difference absolute mean value (DAMV)**	-	0.0256	0.502
**Difference absolute standard deviation value (DASDV)**	-	0.0362	0.471
**Difference variance value (DVARV)**	-	0.0362	0.471
**Enhanced mean absolute value (EMAV)**	-	0.0234	0.510
**Enhanced wavelength (EWL)**	-	0.0162	0.0.540
New zero crossing (FZC)	-	-	0.333
**Kurtosis (KURT)**	-	0.0162	-0.540
**Integrated EMG (IEMG)**	-	0.0214	0.517
**Interquartile range (IQR)**	-	0.0394	0.463
Log CV(LCV)	-	-	
**Log Detector (LD)**	-	0.0428	-0.02
**Log DAMV (LDAMV)**	-	0.0256	0.502
**Log DASDV (LDASDV)**	-	0.0362	0.417
**Log Teager Kaiser energy operator (LTKEO)**	-	0.0394	0.464
**Mean absolute deviation (MAD)**	-	0.0256	0.502
**Mean absolute value (MAV)**	-	0.0256	0.502
**Maximum fractal length (MFL)**	-	0.0362	0.471
Mean (MN)	-	-	0.210
Median (MD)	-	-	-0.241
**Modified mean absolute value (MMAV)**	-	0.0234	0.510
**Modified mean absolute value 2 (MMAV2)**	-	0.0428	0.455
**Mean value of the square root (MSR)**	-	0.0195	0.524
Root mean square (RMS)	-	-	
Standard deviation (SD)	-	-	0.425
Skewness (SKEW)	-	-	0.333
Single square integral (SSI)	-	-	0.425
Absolute value of temporal moment (TM)	-	-	0.433
Variance (VAR)	-	-	0.425
Variance of EMG (VARE)	-	-	0.425
Variance order (VO)	-	-	0.425
**Waveform length (WL)**	-	0.0256	0.502

### 3.3. CoP

#### 3.3.1. Sway profile

Figure 5 highlights the CoP evolution between PD and healthy subjects on two of its main characteristics area and axis length of the sway ellipse. Sway is greater in healthy than PD participants.

The only significant feature (*p* < 0.05) for CoP in the PRE phase is the *Direction Entropy (Nats)*, while the statistically significant ones in the POST are listed below Figure 5 with p-values and cliff delta values listed.

### 3.4. EEG

The topological plots were computed for all frequency bands during the phases of the acquisition. Each of them displays the difference of power spectral density between PD and healthy cohorts, only for the statistically significant electrodes (p ≤ 0.05, represented by a green point in the figure). Theta and alpha bands presented several significant electrodes in different locations of the brain. In the theta band, significant electrodes are found mostly in the temporal lobe (T7, T8, C6, FT7), one in the frontal lobe (AF3) and one in the occipital (PO6). In the alpha band, significant electrodes are found mostly in the temporal lobe (FC5, T7, T8, FT8), one in the parietal lobe (P4) and one in the occipital (PO6). The p-values of each electrode of the theta and alpha bands are shown in Tables 6, 7, comparing the two cohorts in the PRE and POST phases. They highlight the differences in brain activity in the two phases and underline the significant difference in the POST phase of the experiment between the PD and Healthy groups.

**Table 6:** Electrodes for theta band with corresponding *p*-values comparing the two cohorts in POST and PRE phases-bold shows they were significantly different in the POST phase of the experiment.

**Electrodes**	**PRE phase**	**POST phase**
M1	0.124	**0.0326**
T7	0.0740	**0.0169**
T8	0.139	**0.0215**
AF3	0.0947	**0.0383**
C6	0.0784	**0.0247**
PO6	0.0983	**0.0409**
FT7	0.112	**0.0476**

**Table 7:** Electrodes for alpha band with corresponding *p*-values comparing the two cohorts in POST and PRE phases- bold shows they were significantly different in the POST phase of the experiment.

**Electrodes**	**PRE phase**	**POST phase**
FC5	0.0597	**0.0247**
T7	0.122	**0.0187**
T8	0.0703	**0.0247**
P4	0.0596	**0.0391**
PO6	0.276	**0.0247**
FT8	0.0674	**0.0165**

## 4. Discussion

In previous work, we compared the identical PRE and POST phases in order to characterize different cohorts while they performed unassisted standing under conditions of sensory conflict after exposure to the complex postural control task of the moving platform and VR scene. EEG delta and theta power spectrum analysis and EMG activity in the soleus muscle proved to be strong discriminators between groups (Jacob et al., 2022).

In this paper, we use a similar multi-factorial approach to characterize PD and healthy participants on the basis of their postural control response during the BioVRSea experiment. In particular, we compared the balance response after a visual stimulus only–PRE phase: VR visual sea motion simulation; to the balance response obtained after a complete immersive sensory experience–POST phase: VR visual and correspondent motion stimulation.

This study is part of an extensive work in which a larger population is monitored. We have collected data from 324 volunteers (females 183, males 141, general age 33 ± 14). The overall population is between the ages of 18 and 29. Recruiting a larger group of older adults may open a new way to highlight age-related postural control strategies using the same protocol.

### 4.1. Heart rate

Although heart rate per minute appears to increase in subjects with Parkinson's from PRE to POST phase, there is no significant result to underscore the difference in the two cohorts.

### 4.2. Muscle activation

The lower leg muscles are involved in postural and balance control strategies (Loram et al., 2005). Different muscles are involved at different times in two cohorts under the same experiment and this may be pathology dependent. The most significant activity was found in the tibialis anterior on the right leg, with some significant activity in the right soleus and the left tibialis anterior. However, only features obtained from the right tibialis anterior (TA) statistically differentiated the two groups. This may be related to the fact that tibialis anterior is the primary dorsiflexor of the foot, and is critical in gait to lift the foot during the swing phase.

We can hypothesize that in the group of subjects considered in this study (Parkinson's and healthy) has overall right-handed prevalence. This is supported in one of the latest reported studies conducted on the human hand, from which it was stated that the precise prevalence is the right hand in the world population. The prevalence of left-handedness is between 10–20% (Papadatou-Pastou et al., 2020). Thus, we can explain the significant results obtained for the right side through the dominance of the legs.

### 4.3. Center of pressure

A common way to evaluate PD is based on gait analysis with accelerometer and force sensors inside the shoe. Gait analysis has revealed higher frequency values for PD compared to healthy controls. However, until now Parkinson was not assessed by measuring the force in a standing position (Hsieh and Abbod, 2021).

Our results highlight a reduced sway in PD subjects during the task ant that may be related to a multitude of factors. Some diseases, including PD, interfere in the ability to maintain balance. PD patients have less coordination of agonist and antagonist muscles, making it challenging to maintaining stability. They also frequently suffer from limb and axial rigidity that may reduce mobility (Gandolfi et al., 2018). All of those linked to a reduced mobility confidence may account for the reduced sway.

**Figure 6:**
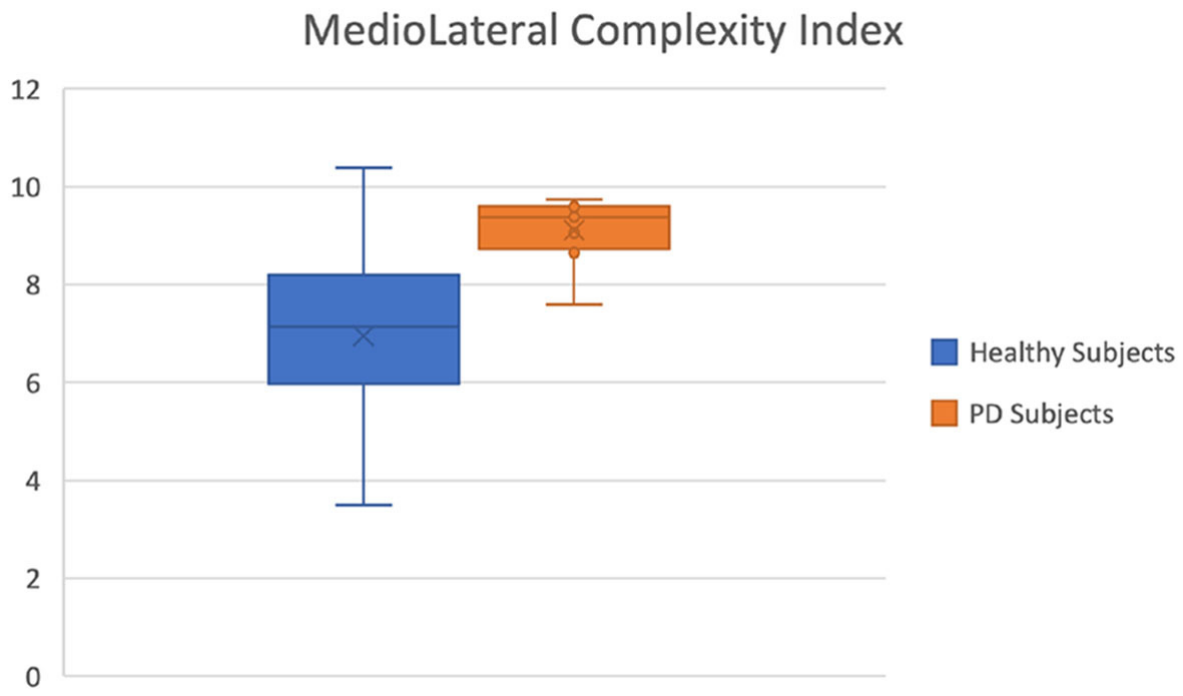
Mediolateral Complexity Index of healthy (blue) and Parkinson (orange) groups.

We found significant differences in the POST phase between the PD and healthy cohorts. This is significant as the POST phase is the stage after a motor stimulus and it can be a good index of pathology progression. The most discriminating feature was found to be the complexity index in the medio-lateral direction as seen in Figure 6. The effect size for this variable is also large (-0.793 - the negative value indicating that most of the higher values for this variable were in the PD group) which is classified as a large effect as per (Vargha and Delaney, 2000) which ranks a delta value greater than 0.42 as a large effect. The mediolateral CI in the PD group is higher than in the healthy cohort, which is contrary to a number of studies which show that the complexity of postural dynamics tends to decrease in disease and aging (Habtemariam et al., 2017; O'Keeffe et al., 2019). It is unclear why the complexity index is higher in the pathological group for our study.

### 4.4. Neural response

The strong involvement of the cerebral cortex in postural control responses to perturbation is well-known, but the correlations with pathologies affecting mobility are still poorly understood (Jacobs and Horak, 2007; Maki and McIlroy, 2007; Papegaaij et al., 2014). Understanding the network of cortical structures involved in a disease such as Parkinson's and how sensory information are processed can be an important step in diagnostics.

Each band can be associated with a particular neural function and in our research, differences in alpha and theta response prove to be statistically significant when comparing between the two groups. The result for alpha band shows that the activity in the healthy subjects is greater than in the Parkinson subjects in both the PRE and POST phases. On the other hand, the theta band shows different behavior depending on the brain area and phase. Theta and alpha bands are involved in the regulation of the posture, in particular when a visual feedback is altered (Kahya et al., 2022). Figure 6 shows the topological plots of absolute PSD for theta and alpha frequency bands. Each row represents the PSD difference of task (PRE and POST). The PSD differences between Parkinson and healthy subjects are compared for significant electrodes with Benjamin-Hochberd FDR procedure.

#### 4.4.1. Theta waves

The theta rhythm is one of the slowest oscillations in the normal waking state, just above the delta rhythm that dominates slow wave sleep. Theta waves are involved in attention and memory processes, especially in memory retrieval episodes (Baars and Gage, 2010). Although the alpha band has been shown to be strongly correlated within postural task conditions, it currently remains less known whether the theta band shows an association with increased postural task difficulty (Kahya et al., 2022). One of the most recent publications on neurophysiology in healthy subjects showed that the theta band has important electrodes located mainly in the parietal scalp, associated with a slight decrease in PSD (Aubonnet et al., 2022). The parietal lobe is activated to plan and process the orientation of the body and sensory information, demonstrating the remarkable role of the theta band in postural strategies.

In our results, parietal activity of the theta band from PRE to POST phase shows a decrease of PSD in healthy subjects, confirming what was found in the work just mentioned. Instead for the frontal lobe, the theta band shows an increased PSD in the healthy group in both tasks. Theta brain rhythms are associated with cognitive and motor functions, and patients with PD would have irregular theta rhythms during lower-limb activations (Singh et al., 2020).

#### 4.4.2. Alpha waves

**Figure 7:**
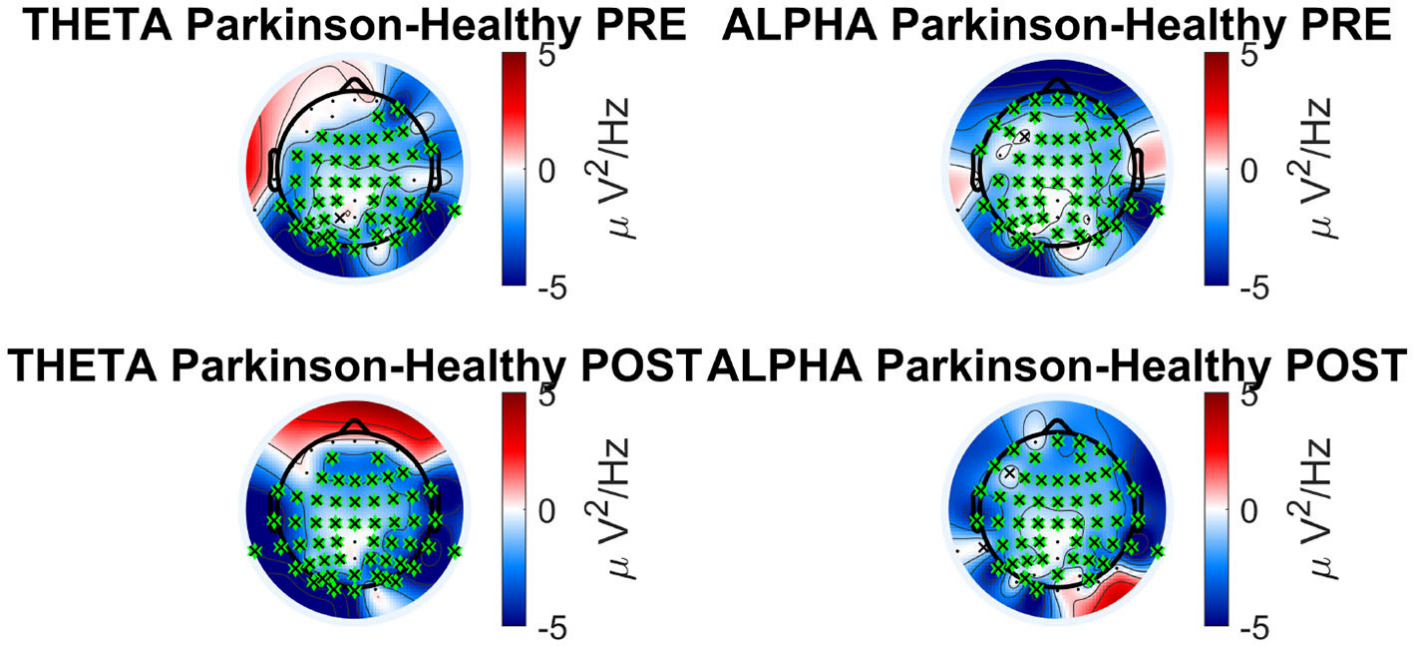
Absolute PSD bands analysis in Parkinson's vs. healthy cohort for theta and alpha frequency band.

Different studies have shown that the performance of a generic balance task results in simultaneous changes in the amplitude of alpha oscillations (Slobounov et al., 2013; Malisova et al., 2017). Alpha activation is associated with cognitive events and has been found to increase during intentional tasks such as mental arithmetic and working memory. Planning actions and their execution also generate alpha (Pfurtscheller, 2003). Therefore, alpha waves play a functional role in human cognition and that it does not represent only an ‘idling rhythm, as many scientists believed until recently. Maintaining balance is an active process and requires constant awareness of any external stimuli. The alpha band has shown interesting results in postural control studies where a decrease in alpha power was associated with an increased task difficulty during upright stance in young adults (Percio et al., 2007; Hülsdünker et al., 2016; Kahya et al., 2022). As the PD group has lower overall alpha power (as seen in Figure 7), this may indicate higher demand to cope with the balance task compared to the healthy group. In another publication on the neurophysiology of healthy subjects using the BioVRSea experiment (Aubonnet et al., 2022), the alpha band is important for balance control across the whole scalp. Our study is consistent with these results.

Both the bands (theta and alpha) can be considered parameters to discriminate PD subjects during a complex postural control task and confirm the activation of the frontal, parietal and visual lobe in healthy subjects, underlining the difficulty the PD group experiences when making postural adjustments.

## 5. Conclusions and limitations

Our paper confirms that a comprehensive multi-factorial approach (our unique BioVRSea paradigm) is useful in discriminating early PD subjects. In addition, the ability to look at multiple parameters at once introduces the ability to further analyse the correlation and timing of our set of specific features changes. In fact, there are no previous multi-metric experiments, such as BioVRSea, and there are no experiments with larger patient cohorts, therefore a larger study is foreseen to provide more definitive conclusion. Several neural and motor strategies difference have been highlighted, and are in line with known literature.

It is also worth providing some elements for consideration, specifically in the PD diagnosis. Firstly, the 'early stage classification' has no absolute value, it is a subjective evaluation and may suffers from a degree of variability. This influences the time of the diagnosis, which, in turn, may also present early stage cohorts with non-homogeneous symptoms and impairment. This BioVRsea paradigm seems very promising for comprehensive quantitative assessments and may pave the way for highlighting the most relevant features in the specific motor diseases analyzed. This may favor the introduction of less complex quantitative analyses, specifically for clinical operators, to replicate only the most poignant aspects of our paradigm. Our results suggest that a simpler experimental design including concurrent EEG, bilateral lower limbs EMG and CoP analyses with a balance challenge (not necessarily VR led) can discriminate early stage PD and has the potential to stratify further stages of the pathology.

Postural control alteration is one of the major risk factors to facilitate the occurrence of falls in the elderly. The frailty index, which is the expression of the health status of older individuals, is related to falls (Tornero-Quinones et al., 2020; Taguchi et al., 2022). Higher levels of frailty indicate the presence of chronic diseases (Vinik et al., 2017), such as diabetes, chronic pain, and polypharmacy. Taken separately, those clinical conditions have been established to change the postural control and increase falls (Efstathiou et al., 2022; Rasmussen et al., 2023). We speculate that BioVRSea protocol may be added to the routine assessment of individuals with a high frailty index to identify early postural control deficit and address early intervention to prevent falls. Furthermore, individuals with other neurodegenerative disorders (i.e., multiple sclerosis - Comber et al., 2018; Molhemi et al., 2021 - or dementia - Nyman et al., 2019; Chepisheva, 2023), or neurological conditions (i.e., stroke - Chen et al., 2016 - or brain injury - Perez et al., 2020) may find advantage in the use BioVRSea protocol to explore the postural control alterations.

BioVRSea is an innovative model to analyze postural control response that can be used for different clinical applications. It may also prove useful to classify other related diseases and conditions of movement disorders, such as Progressive Supranuclear Palsy (PSP) or Huntington's disease (Porciuncula et al., 2020) and many other disorders that involve changes in postural control such as loss of balance and slow movement, where a similar performance can be expected. Having new collaborations with different disease categories will add more value and amplify the research.

## Data availability statement

The raw data supporting the conclusions of this article will be made available by the authors, without undue reservation.

## Ethics statement

The studies involving human participants were reviewed and approved by Icelandic National Bioethics Committee (Application no: 17-183-S1). The patients/participants provided their written informed consent to participate in this study.

## Author contributions

The idea for the paper was conceived by PG, HP, and VM. FP, LG, DJ, and SP processed the raw data from EEG, EMG, CoP, and HR and made the figures and tables. CG performed statistical analysis on the processed data. FP, LG, DJ, CG, PG, GD, and AF contributed to the text for the manuscript. All authors contributed to the article and approved the submitted version.
